# Highly Sensitive and Selective Fluorescent Detection of Gossypol Based on BSA-Stabilized Copper Nanoclusters

**DOI:** 10.3390/molecules24010095

**Published:** 2018-12-28

**Authors:** Shuangjiao Xu, Kehai Zhou, Dan Fang, Lei Ma

**Affiliations:** 1 State Key Laboratory of Cotton Biology, Institute of Cotton Research of CAAS, Anyang 455000, China; mhsxsj@126.com (S.X.); zhoukehai@caas.cn (K.Z.); holiday_8320@163.com (D.F.); 2 Research Base, State Key Laboratory of Cotton Biology, Zhengzhou University, Zhengzhou 450001, Henan, China

**Keywords:** copper nanoclusters, gossypol, fluorescence, probe

## Abstract

In this paper, fluorescent copper nanoclusters (NCs) are used as a novel probe for the sensitive detection of gossypol for the first time. Based on a fluorescence quenching mechanism induced by interactions between bovine serum albumin (BSA) and gossypol, fluorescent BSA-Cu NCs were seen to exhibit a high sensitivity to gossypol in the range of 0.1–100 µM. The detection limit for gossypol is 25 nM at a signal-to-noise ratio of three, which is approximately 35 times lower than the acceptable limit (0.9 µM) defined by the US Food and Drug Administration for cottonseed products. Moreover, the proposed method for gossypol displays excellent selectivity over many common interfering species. We also demonstrate the application of the present method to the measurement of several real samples with satisfactory recoveries, and the results agree well with those obtained using the high-performance liquid chromatography (HPLC) method. The method based on Cu NCs offers the followings advantages: simplicity of design, facile preparation of nanomaterials, and low experimental cost.

## 1. Introduction

Gossypol is a yellow polyphenolic compound that occurs naturally in certain species of cotton plants of the family Malvaceae, especially in their seeds. Cottonseed is either fed to animals as whole seeds, or the edible oil is extracted for human consumption while the resulting meal is used as a feed supplement for livestock [1]. However, gossypol may produce adverse physiological effects [2]. The compound can be toxic to all animals in sufficient quantities and has been used in small controlled doses as a male contraceptive in humans. It can also cause temporary male infertility [3]. The safe upper limit of gossypol in cotton products is fixed at 0.9 µM in the USA and 0.4 µM in China. Thus, the development of a facile, robust, and sensitive method for the determination of gossypol is necessary. Several techniques have been used for the determination of gossypol, such as high-performance liquid chromatography (HPLC) [1,4,5,6], UV spectrophotometry [7,8], capillary electrophoresis (CE) [9], and near infrared (NIR) spectroscopy [10,11]. However, these methods are limited by high costs, sophisticated chemical syntheses, or poor sensitivity, which restrict their practical applications.

Fluorescence methods have proven to be powerful optical techniques for the detection of trace analytes. To date, few reports have been published regarding the fluorescence detection of gossypol [12,13]. Furthermore, for the type of method described in these reports, complex chemical modification or strict reaction conditions are needed. Recently, metal nanoclusters (NCs) consisting of several atoms have sparked considerable interest as novel fluorescent markers owing to their ultra-small size, biocompatibility, and water solubility [14,15,16]. Among these metal NCs, protein-protected Au and Ag NCs are currently the most intensively explored [17,18,19,20,21,22]. However, because the cost of gold and silver precursors is very high, the exploration of new, cheap, and biocompatible metal nanoclusters is of great value. Copper NCs fit these characteristics [23]. In addition, the application of protein-coated fluorescent metal NCs in analysis mainly relies on the quenching of NC fluorescence through interactions between the cluster and ions (Hg^2+^, Cu^2+^, CN^−^) [18,21,24] or through oxidation of the Au-S bonds between the cluster and protein by hydrogen peroxide (H_2_O_2_) [25]. However, gossypol has been reported to form a stable complex with bovine serum albumin (BSA) which induces concentration-dependent conformational changes in the vicinal groups of the binding sites [13,26]. This phenomenon provides new insight into the direct fluorescence regulation of BSA-stabilized Cu NCs (BSA-Cu NCs) by gossypol, which is valuable for the establishment of new methods.

In this work, we demonstrate a new strategy for developing the first turn-off fluorescent method for the determination of gossypol using BSA-Cu NCs as the fluorescent indicator. This method combines the sensitivity of a fluorescence technique with the high affinity of BSA for gossypol. The proposed method is selective and sensitive and was successfully applied to the detection of gossypol in meal and oil samples of cottonseed. 

## 2. Results and Discussion

### 2.1. Characterization of BSA-Cu NCs

**Figure 1:**
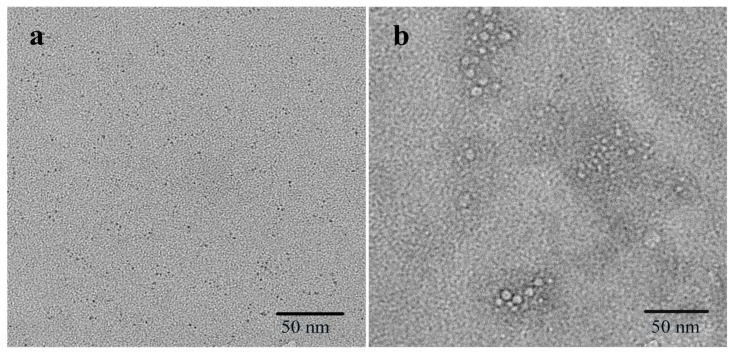
Transmission electron microscopy (TEM) images of the bovine-serum-albumin-stabilized Cu nanoclusters (BSA-Cu NCs) in the absence (**a**) and presence of 100 μM gossypol (**b**).

**Figure 2:**
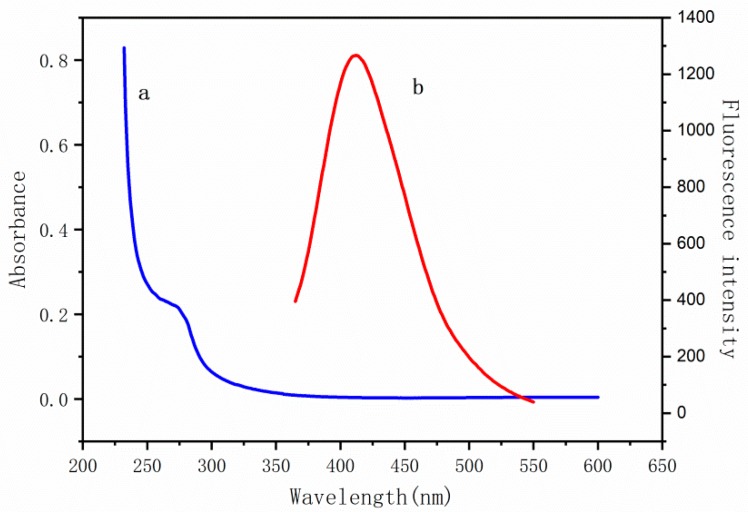
Absorbance (**a**) and fluorescence emission (**b**) spectra of the BSA-Cu NCs.

**Figure 3:**
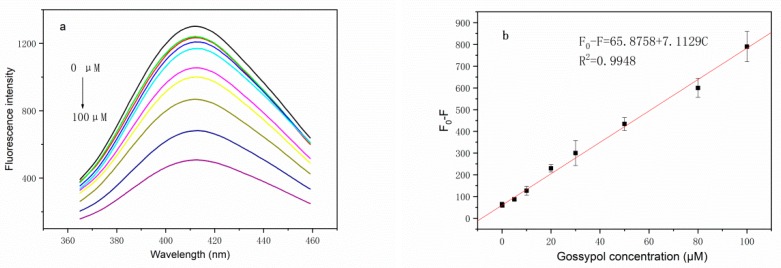
(**a**) Fluorescence spectra of the BSA-Cu NCs in the presence of different gossypol concentrations. Conditions: BSA-Cu NCs, 21 mg L^−1^; Tris-HCl solution, 0.1 mM; pH = 7.4. The concentrations of gossypol were 0.00, 0.01, 0.10, 5.00, 10.00, 20.00, 30.00, 50.00, 80.00, and 100.00 μM. (**b**) Linear relationship between the decrease in fluorescence intensity and the concentration of gossypol. Error bars were estimated from three replicate detections.

Figure 1a shows typical transmission electron microscopy (TEM) images of the as-synthesized BSA-Cu NCs, which appear as spherical monodisperse clusters with sizes of approximately 2 nm. The TEM images reveal that aggregation of the Cu NCs occurred following the addition of gossypol (Figure 1b). The optical properties of an aqueous solution of the BSA-Cu NCs were investigated by UV-visible and fluorescence spectroscopies. When considering the UV-vis absorption spectrum (Figure 2a), the Cu NCs are clearly relatively small, which is consistent with the TEM results. Upon excitation at 320 nm, the BSA-Cu NCs showed an emission band centered at 400 nm (Figure 2b). The character of the spectrum was consistent with that reported in the literature [27,28], which suggests the successful preparation of BSA-Cu NCs. The fluorescence response of the BSA-Cu NCs to gossypol is shown in Figure 3a. The emission intensity of the BSA-Cu NCs decreased by 66% after the addition of 100 µM of gossypol, indicating that the BSA-Cu NCs could potentially be used as a fluorescent probe for the detection of gossypol.

### 2.2. Optical Stability of the BSA-Cu NCs

**Figure 4:**
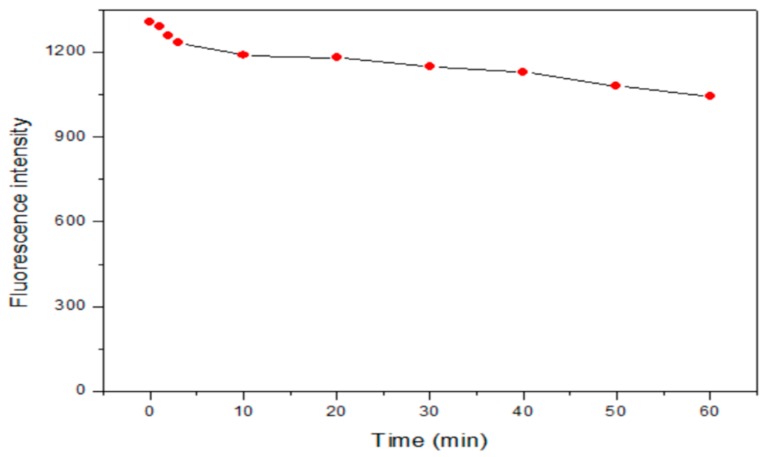
Photostability study of the BSA-Cu NCs.

To explore the photostability of the obtained BSA-Cu NCs, the BSA-Cu NCs were exposed to UV irradiation with a wavelength of 365 nm for 60 min. Most organic dyes will suffer from light degradation over this length of time. As shown in Figure 4, a decrease in fluorescence of only 20% demonstrated that the BSA-Cu NCs possess great photostability, which makes them a promising candidate for continuous tracking studies over a long period of time. The slight fluorescence quenching of the Cu NCs can be attributed to conformational changes in the BSA on the surface of the NCs, which may lead to agglomeration or the disintegration of nanoparticles. A similar case has been reported by Georgina’s group [29].

### 2.3. Detection of Gossypol by the Fluorescent BSA-Cu NCs

Figure 3b shows the dependence of the fluorescence intensity of the BSA-Cu NCs on the concentration of gossypol. Fluorescence intensity decreased linearly with increasing concentrations of gossypol from 0.1 µM to 100 µM. The relative standard deviations (RSDs) were 4.8% and 3.6% for six repeated measurements of 1 µM and 10 µM gossypol, respectively. The limit of detection (LOD) was 25 nM at a signal-to-noise ratio of three. Compared with other reported gossypol detection methods as shown in Table S1, this fluorescence method is more sensitive and much simpler.

**Figure 5:**
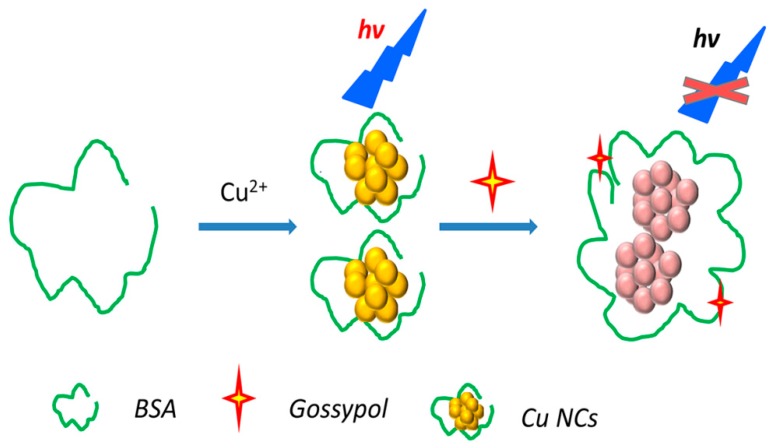
Scheme illustrating the fluorescence sensing mechanism.

The observed fluorescence quenching phenomenon likely results from a strong interaction between gossypol and the BSA-Cu NCs. The formation of a complex between gossypol and BSA with a 1:1 stoichiometry has been reported to be stable for long periods (K_d_ = 2.7 × 10^3^ M^−1^) [30]. This binding complex appears to be stabilized by hydrophobic and hydrogen-bonding interactions [13]. Additionally, conformational changes were observed in the BSA that depended on the total concentration of gossypol added [12]. The protective ligand is known to play a major role in the fluorescence of metal NCs as the disruption of functional groups or the conformation of the protective ligand has been reported to substantially influence the optical properties of NCs [31]. In the absence of gossypol, the Cu NCs are monodispersed (Figure 1a) and have a mean diameter of approximately 2.0 nm, whereas aggregation of the Cu NCs is clearly observed in the presence of gossypol (Figure 1b). These results illustrate that Cu NCs could interact with gossypol to induce their aggregation. The detailed detection procedure is illustrated in Figure 5. The BSA-Cu NCs exhibited an intense emission band at 400 nm. In the presence of gossypol, a stable complex formed between gossypol and BSA, and the subsequent aggregation of NCs led to a decrease in the fluorescence intensity.

**Figure 6:**
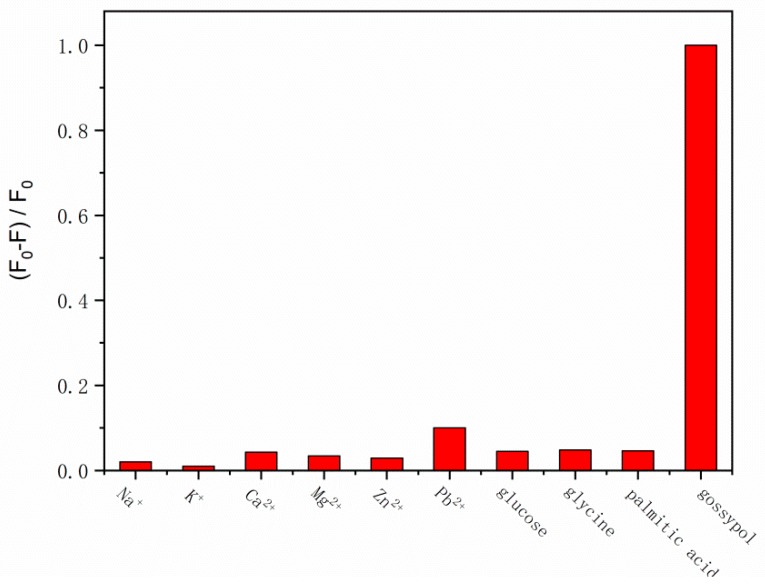
Selectivity of the BSA-Cu NCs over potentially interfering substances. Gossypol: 1 µM; Na^+^, K^+^, Ca^2+^, Mg^2+^, and Zn^2+^: 100 µM; glucose, glycine, and palmitic acid: 65 µM.

To determine gossypol concentrations in real samples such as cottonseed meal and oil, potential interfering substances, including common ions, glucose, lactose, and some proteins, were investigated to evaluate the selectivity of the proposed method under the same conditions. The tolerance ratio mentioned here is the ratio of the measured concentration to the actual concentration. As shown in Figure 6, most of the interfering substances, including Na^+^, K^+^, Ca^2+^, Mg^2+^, Zn^2+^, glucose, glycine, and palmitic acid, exhibited tolerance ratios at least 65 times greater than that of gossypol, while the changes in fluorescence intensity were less than 5%, indicating that the interfering substances listed above could only minimally interfere with the detection of gossypol. However, slight quenching of the luminescence was observed in the presence of Pb^2+^ ions, which may be due to the formation of a complex between BSA and the Pb^2+^ ions. Nevertheless, the ratio of the fluorescence intensity change induced by Pb^2+^ ions to that of gossypol was much lower than the allowed ratio. The results indicate that the method shows good selectivity and sensitivity to gossypol and is capable of detecting gossypol in real samples.

### 2.4. Detection of Gossypol in Cottonseed Samples

To evaluate the success of our proposed method on real samples, we investigated the feasibility of assays in the fluorescence detection of gossypol in cottonseed. The measured concentrations of gossypol in dilute samples are listed in Table S2. The analyses were performed in parallel with HPLC for comparison purposes, and a standard deviation of < 5% between the results of the two methods was determined. The good recoveries (from 80.6% to 106.9%) of known amounts of gossypol added to the cottonseed samples definitively demonstrate the accuracy and reliability of the present method for detecting gossypol in practical applications.

## 3. Materials and Methods

### 3.1. Materials 

BSA, H_2_O_2_, copper sulfate (CuSO_4_), and sodium hydroxide (NaOH) were obtained from Shanghai Sangon Biotechnology Co., Ltd. (Shanghai, China). Gossypol, glucose, palmitic acid, and glycine were purchased from Sigma-Aldrich Chemical Co. (St. Louis, MO, USA). Ultra-pure water was used throughout the experiments.

### 3.2. Apparatus

Fluorescence measurements were performed on a Hitachi Model F-2500 fluorescence spectrophotometer (Hitachi, Tokyo, Japan). The emission spectra were recorded upon excitation at 320 nm. The slits for excitation and emission were both set at 10.0 nm. Steady-state absorption measurements were carried out on a Beckman DU-800 UV-vis spectrophotometer (Beckman Coulter, Inc., Fullerton, CA, USA) by scanning in the range 200–600 nm. Transmission electron microscopy (TEM) images were collected on a Tecnai G2 F20 transmission electron microscope (FEI, Hillsboro, FL, USA) operating at 200 kV.

### 3.3. Synthesis of BSA-Cu NCs

In a typical preparation process [28,29], 4 mL of a CuSO_4_ solution (20 mM) was added into 20 mL of a BSA solution (10 mg mL^−1^). After stirring for 5 min, the pH of the solution was slowly adjusted to 12 using a NaOH solution (1 M). Then, 15 mL of an H_2_O_2_ solution (0.1 M) was added under vigorous agitation and the mixed solution was heated at 55 °C for 1 h to generate the BSA-Cu NCs. The product was stored in a refrigerator at 4 °C until use.

### 3.4. Fluorescence Detection of Gossypol

A stock solution of gossypol (10 mM) was freshly prepared before use. Gossypol solutions with varying concentrations were obtained by serial dilution of the stock solution. For the gossypol detection experiments, 50 µL of gossypol solutions with different concentrations was added into 50 µL of the prepared BSA-Cu NCs solution. Then, 200 µL of Tris-HCl (0.1 mM, pH = 7.4) was added to give a final volume of 300 µL and the solution was mixed by a vortex mixer for a few seconds. After incubation for 10 min at 37 °C, fluorescence intensities were immediately recorded in the wavelength range from 360 nm to 460 nm unless otherwise noted.

### 3.5. Gossypol Determination in Cottonseed Meal and Oil

Cottonseed samples were prepared according to GB 5009.148-2014. Briefly, a cottonseed meal/oil sample (0.1 g) was weighed into a 10 mL centrifuge tube. Then, ethanol (5 mL) was added and the mixture shaken vigorously. The mixture was allowed to separate overnight in a refrigerator and was then centrifuged at 4000 rpm for 10 min. A 10 µL aliquot of the upper organic layer was transferred to a 10 mL volumetric flask, diluted to 10 mL with ethanol, and finally used for detection.

## 4. Conclusions

In summary, a sensitive turn-off fluorescence method for detecting gossypol based on BSA-Cu NCs has been developed. We have demonstrated that the mechanism is based on the quenching of fluorescence intensity due to the formation of a complex between gossypol and the BSA on the surface of the NCs which induces aggregation of the NCs. The proposed method displays high sensitivity, a low detection limit, and good performance in the determination of real samples without the requirement of complicated modification procedures and expensive instruments. To the best of our knowledge, this is the first report on the design of a fluorescence method for the detection of gossypol, and this method provides potential ways to apply Cu NCs in different sensors. 

## Supplementary Materials

The Supplementary Materials are available online.
