# Supplementary material for: Highly Sensitive and Selective Fluorescent Detection of Gossypol Based on BSA-Stabilized Copper Nanoclusters
Source: Molecules. 2018 Dec 28;24(1):95. doi: 10.3390/molecules24010095 (PMC6337446; doi:10.3390/molecules24010095)
Supplement: Supplementary file 1 [file molecules-24-00095-s001.pdf]

Supporting information for the manuscript entitled:

Highly Sensitive and Selective Fluorescent Detection of Gossypol Based on BSA-stabilized Copper Nanoclusters

Shuangjiao Xu[1], Kehai Zhou[1], Dan Fang[1], Lei Ma[1, 2]*

[1] State Key Laboratory of Cotton Biology, Institute of Cotton Research of CAAS, Anyang 455000, China

[2] Research base, State Key Laboratory of Cotton Biology, Zhengzhou University, Zhengzhou, Henan 450001, China

* Correspondence: malei@caas.cn; Tel.: 86-372-2562278

Table S1 Comparison of the analytical performance of the proposed method with others

Method	Linear range (μg mL^{-1})	Detection limit (μg mL^{-1})	References
HPLC-UV	0.05-2.0	0.04	[1]
Ultrasonic-HPLC	4.4-35	/	[2]
HPCE	10-100	1.00	[3]
HPLC-EC	0.001-0.2	0.0025	[1]
Fluorescence	0.05-50	0.012	This paper

Table S2 Determination of gossypol content in real samples by standard addition method (n=3)

Samples	Added (ug mL^{-1})	Found (ug mL^{-1})	Recovery (%)	RSD (%)
Cotton oil 1	0	0. 640	–	4.0
	5	5.706	101.3	4.2
	10	9.583	95.2	3.9
Cotton oil 2	0	0.197	–	3.7
	5	5.490	106.9	4.6
	10	10.010	98.1	4.2
Cotton seed 1	0	9.300	–	4.1
	1	10.231	93.1	3.5
	5	14.244	98.9	3.1
Cotton seed 2	0	4.620	–	3.7
	0[a]	4.534[a]	–	1.9[a]
	1	5.426	80.6	2.9
	1[a]	5.588[a]	96.8[a]	2.0[a]
	5	9.856	104.7	3.2
	5[a]	9.650[a]	100.6[a]	2.2[a]

[a] HPLC method

References

[1] K.J. Lee, K. Dabrowski, High-performance liquid chromatographic determination of gossypol and gossypolone enantiomers in fish tissues using simultaneous electrochemical and ultraviolet detectors, J Chromatogr B Analyt Technol Biomed Life Sci, 2002,779, 313-9.

[2] Tian, X. H., Xie, J., X., Determination of free gossypol content in cottonseed by reversed phase-high performance liquid chromatography with ultrasonic extraction, J Analytical Science, 2012,28, 435-438.

[3] Guo, Y. J., Mollemin, D.,Ren, L.J.,Yao, J., Determination of free gossypol content in coyyonseed oil by high efficiency capillary electrophoresi, J Xinjiang Medical University, 2017,40,1074-1080.
